# DDX17 and viral infection

**DOI:** 10.1080/21505594.2025.2602269

**Published:** 2025-12-10

**Authors:** Yuting Cheng, Ruohan Wang, Anping Wang, Zhi Wu, Wenfeng Jia, Huipeng Lu, Qingguo Wu, Shanyuan Zhu

**Affiliations:** a Engineering Technology Research Center for Modern Animal Science and Novel Veterinary Pharmaceutic Development, Jiangsu Key Laboratory of Veterinary Bio-Pharmaceutical High Technology Research, Jiangsu Agri-Animal Husbandry Vocational College, Taizhou, China; b Department of Biopharmaceuticals, College of Medicine and Chemistry & Chemical Engineering, Taizhou University, Taizhou, China

**Keywords:** DDX17, viral infection, RNA helicase, replication, antiviral target

## Abstract

DDX17 (DEAD-box RNA helicase 17) is an essential RNA helicase and regulatory ATPase in host cells, extensively involved in various cellular processes during viral infections, such as RNA splicing, transcriptional regulation, and post-transcriptional modification. DDX17 exhibits dual functionality in viral infections: it enhances the stability, packaging, and replication of viral RNA through interactions with viral ribonucleoprotein complexes, as evidenced in infections caused by influenza viruses and Hantaan virus (HTNV). Conversely, DDX17 can inhibit viral proliferation by disrupting viral RNA metabolism, as observed in hepatitis B virus (HBV) and Epstein-Barr virus (EBV) infections, where it suppresses replication by modulating viral RNA decapping and degradation. The dual role of DDX17 provides novel insights into host-virus interactions while also highlighting its significant potential as an antiviral therapeutic target. These findings are expected to establish a theoretical foundation for related research and offer valuable references for developing novel antiviral strategies.

## Introduction

**Figure 1.**
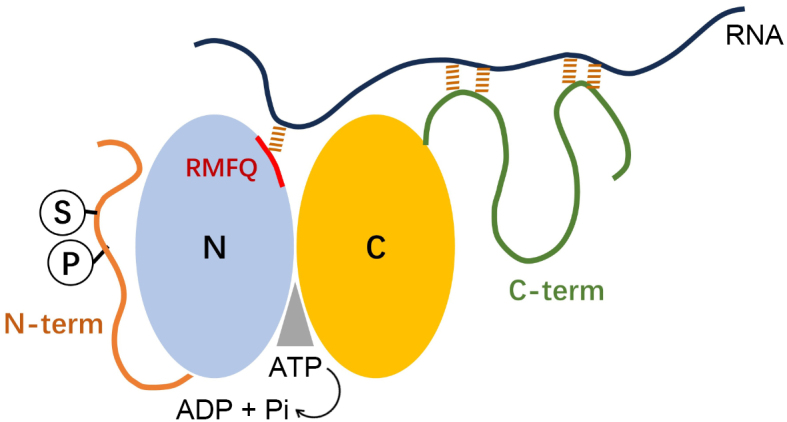
Mechanistic Model for DDX17 function.

DEAD-box RNA helicase 17 (DDX17) is a member of the DEAD-box protein family, first identified by Gâbor M. Lamm et al. in 1996, named for its conserved amino acid sequence Asp-Glu-Ala-Asp (D-E-A-D) [1]. As an RNA-binding protein, DDX17 recognizes and binds to various RNA molecules, participating in multiple critical biological processes, including DNA repair, histone modification, and miRNA regulation [2–4]. Like all DEAD-box helicases, DDX17 plays diverse roles in RNA metabolism, such as RNA splicing, translation, degradation, and ribosome biogenesis [5,6]. The DDX17 mRNA can be alternatively translated into two isoforms, p72 and p82, which share nearly identical properties, leading most studies to overlook their functional distinctions [7]. In host cells, DDX17 interacts with HDAC1 (Histone Deacetylase 1), ERα (estrogen receptor α), and U1 snRNP to regulate gene transcription, ribosome biogenesis, and RNA splicing [8,9]. Additionally, DDX17 acts as an auxiliary factor for the microprocessor complex, mediating the recognition and processing of primary miRNA (pri-miRNA) into mature miRNA [10] (Figure 1).

Viruses are small, acellular entities with simple structures that contain either DNA or RNA and must parasitize live cells to replicate. Increasing evidence has demonstrated a close relationship between viruses and DDX17. DDX17 has a dual influence on viral infection: on one hand, it promotes the replication of certain viruses (e.g. HIV, influenza virus, and Hantaan virus) through interactions with viral proteins or RNA [11–13]. For instance, DDX17 enhances the replication capacity of viruses by regulating the synthesis and processing of viral RNA [14]. On the other hand, DDX17 can also function as an antiviral factor in the infection of certain viruses (e.g. HBV and EBV) by inhibiting viral replication [15,16]. It does this by interfering with the modification and decapping processes of viral RNA, reducing its stability and thereby limiting viral replication [16]. Furthermore, DDX17 serves as an auxiliary factor for zinc-finger antiviral protein (ZAP)-mediated degradation of retroviral RNA, directly contributing to antiviral functions [15].

DDX17 is a multifunctional RNA helicase that plays a crucial role in RNA metabolism and antiviral immune responses. This review systematically summarizes and analyzes the diverse functions and specific mechanisms of DDX17 during viral infections. By participating in RNA splicing, transcriptional regulation, and post-transcriptional modification, DDX17 emerges as a pivotal host factor. Understanding the mechanisms underlying DDX17’s interactions with various viruses offers new insights into host-virus interplay. These findings highlight the significance of DDX17 in fundamental virology.

This paper aims to uncover the unique roles of DDX17 in diverse viral infections through a comprehensive review of existing studies and explores its prospects as a therapeutic target for antiviral interventions. The intricate interactions between DDX17 and various viruses provide a fresh perspective on the mechanisms of viral infections and lay a theoretical foundation for developing innovative antiviral strategies. The insights presented in this paper contribute to advancing our understanding of DDX17 in the field of virology and may offer groundbreaking solutions to alleviate the global burden of viral pandemics.

## Molecular structural features of DDX17

**Figure 2.**
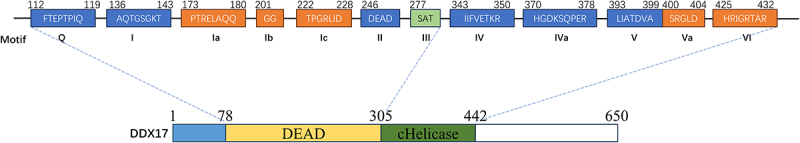
Schematic representation of DDX17 structure.

DDX17 is primarily located in the nucleus of HeLa cells (human cervical adenocarcinoma-derived cell line) and acts as an RNA-dependent ATPase and ATP-dependent RNA helicase, playing a vital role in RNA metabolism. The core region of DDX17 consists of nine conserved motifs, including motifs Q, I, Ia, Ib, II, III, IV, V, and VI, which collectively maintain the functional integrity of DDX17 in RNA metabolism [17]. Motif Q is responsible for sensing and binding ATP, which is hydrolyzed into ADP + inorganic phosphate (Pi) to release energy for RNA unwinding [18]. Motifs I and II are widely present in various NTPases, facilitating the binding of ATP and associated Mg^2+^ [19]. Motifs II and III are essential for ATPase activity and work in concert with motifs I and VI to form an ATP-binding pocket, ensuring the correct positioning of residues required for hydrolysis. Motif IV is considered key for binding single-stranded RNA (ssRNA) and acts as a functional link between motifs IV and V, while motifs Ia, Ib, IV, and V are involved in substrate RNA binding [20]. The core region formed by these motifs consists of two flexibly linked RecA-like domains, referred to as domain 1 (D1) and domain 2 (D2), which play crucial roles in the functional execution of DDX17 [21,22] (Figure 2).

D1 is primarily involved in ATP binding and hydrolysis and is indispensable for helicase activity. D2 stabilizes the domain structure and expands its RNA-binding surface [21]. When the two domains are in a closed state, they create a gap in which motifs form an active ATPase binding site, leading to ATP hydrolysis and energy release [23]. Flanking the conserved core region of DDX17 are N-terminal and C-terminal domains that interact with RNA substrates or auxiliary factors, thereby targeting and regulating the helicase activity of DDX17 [21]. Unlike other DEAD-box RNA helicases, DDX17 features a unique N-terminal domain containing repeated RGG sequences and a C-terminal domain rich in serine and glycine, terminating with a proline-rich region. The core region of DDX17 shares 90% sequence homology with that of DDX5, suggesting similar functions in cellular processes such as transcriptional regulation, RNA splicing, and ribosome biogenesis. However, due to only 30% homology between the C-terminal domains of DDX17 and DDX5, there remain significant differences in certain biological functions [6].

### Physicochemical properties of DDX17 protein

According to the predictions from the ProtParam software, the molecular formula of the DDX17 protein is C_3509_H_5518_N_1026_O_1077_S_30_, specific to human DDX17 and potentially varying across species, encoding 731 amino acids with a theoretical molecular weight of 80, 272.44 Da and an isoelectric point of 8.62. It does not contain Pyl (O) or Sec (U). The total number of positively charged amino acid residues (Arg + Lys) in DDX17 protein is 91, while the total number of negatively charged residues (Asp + Glu) is 84 (Table 1). The instability index of this protein is 48.55 ( >40), indicating that the protein is unstable; the hydrophobicity index is 65.70.

**Table 1.** Amino acid composition of DDX17 protein.

Amino acids	Number	Percentage/%	Amino acids	Number	Percentage/%
Ala (A)	59	8.1	Arg (R)	59	8.1
Asn (N)	23	3.1	Asp (D)	43	5.9
Cys (C)	12	1.6	Gln (Q)	39	5.3
Glu (E)	41	5.6	Gly (G)	73	10.0
His (H)	11	1.5	Ile (I)	32	4.4
Leu (L)	50	6.8	Lys (K)	32	4.4
Met (M)	18	2.5	Phe (F)	23	3.1
Pro (P)	59	8.1	Ser (S)	45	6.2
Thr (T)	43	5.9	Trp (W)	5	0.7
Tyr (Y)	29	4.0	Val (V)	35	4.8

### Hydrophobicity analysis of DDX17 protein

**Figure 3.**
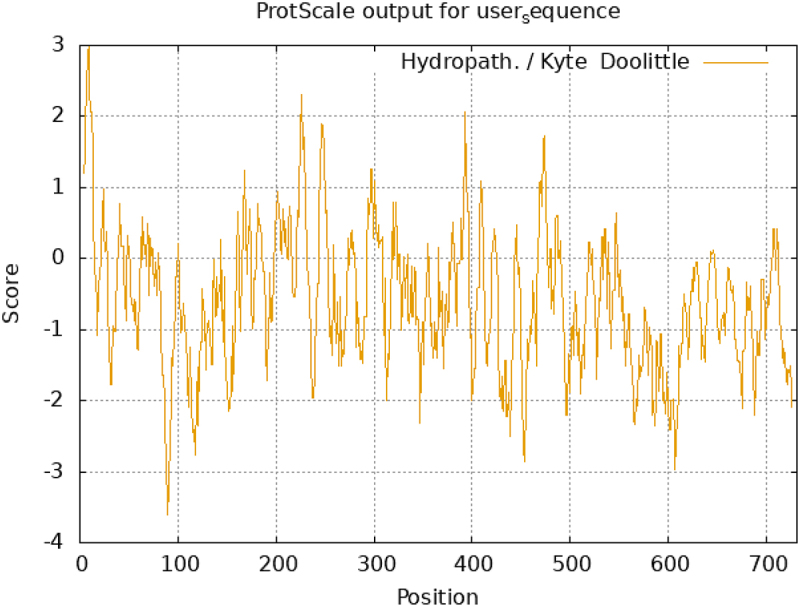
DDX17 protein hydrophilicity analysis.

Using the ProtScale tool to analyze the distribution of hydrophobic amino acid residues in DDX17 protein, the maximum hydrophobicity was observed at the 9th amino acid with a value of 3.000, while the minimum hydrophilicity was at the 89th and 90th amino acids with values of −3.600, indicating that the protein is hydrophilic (Figure 3).

### Prediction of transmembrane structure, signal peptide, and subcellular localization of DDX17 protein

**Figure 4.**
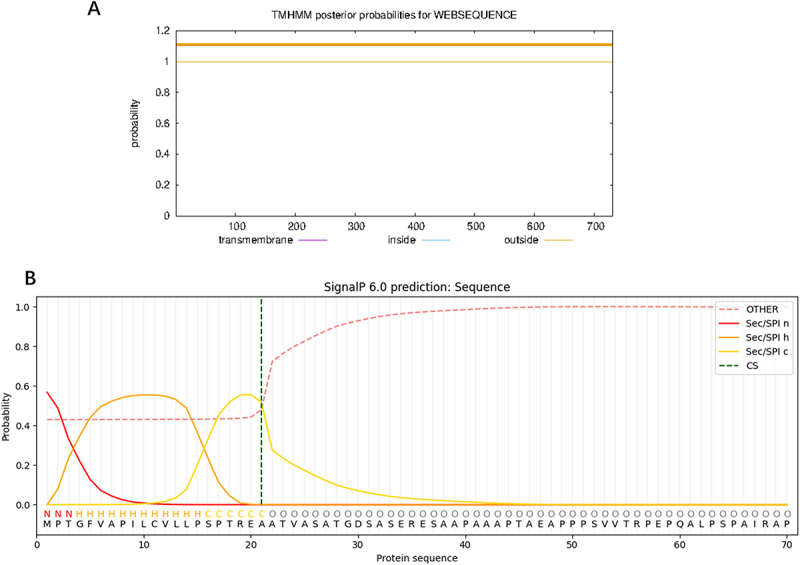
Prediction of DDX17 protein transmembrane region (A) and signaling peptide (B).

The TMHMM predictions indicate that DDX17 protein lacks transmembrane regions and is distributed entirely outside the cell membrane (Figure 4(A)). The SignalP 6.0 predictions for DDX17 protein signal peptides show maximum values for C, Y, and S at 0.266, 0.378, and 0.669, respectively, indicating that DDX17 protein possesses a signal peptide (Figure 4(B)). Subcellular localization analysis reveals that DDX17 protein is primarily located in the cell nucleus [7].

### Prediction of secondary and tertiary structures of DDX17 protein

**Figure 5.**
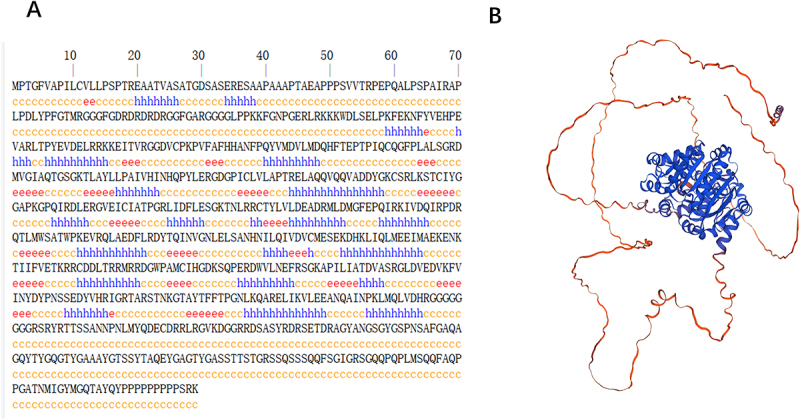
Prediction of DDX17 protein secondary structure (A), tertiary structure (B).

The secondary structure prediction of DDX17 protein using SOPMA indicates that α-helices account for 24.01%, extended strands for 11.39%, and random coils for 64.61% (Figure 5(A)). The tertiary structure prediction using SWISS-MODEL reveals that the tertiary structure of DDX17 protein mainly consists of α-helices and random coil structures, consistent with the predictions of its secondary structure (Figure 5(B)).

## DDX17 in viral infections

DDX17 influences viral infections by modulating viral RNA synthesis and interacting with the host’s antiviral defenses. On one hand, DDX17 facilitates viral replication and propagation in cases such as HIV, influenza virus, and Hantaan virus, where it enhances the packaging and stability of viral RNA. Conversely, DDX17 can inhibit viral replication, as seen with hepatitis B and Epstein-Barr viruses, by interfering with viral RNA modifications and stability. This review will explore the “love-hate” relationship of DDX17 with various viral infections, summarizing its roles and mechanisms.

### DDX17 and sindbis virus (SINV)

SINV is a single-stranded positive-sense RNA virus with an envelope, representing the genus Alphavirus [24]. Its genome is approximately 12 kb, organized into two open reading frames (ORFs) separated by a short non-coding region [25]. The first ORF encodes four non-structural proteins (nsp1-nsp4) involved in viral RNA replication and transcription, while the second ORF encodes structural proteins (C, E1, E2, E3, and 6K) necessary for virion assembly [26,27]. Studies have shown that DDX17 is a critical regulator during SINV infection. Knockdown of DDX17 significantly inhibits SINV replication. DDX17 and DDX5 relocalize from the nucleus to the cytoplasm upon infection and interact with the viral capsid protein, enhancing RNA replication, protein synthesis, and virion formation. Simultaneous inhibition of DDX17 and DDX5 exerts an accumulative effect in suppressing SINV infection [28].

### DDX17 and Zika Virus (ZIKV)

ZIKV is an enveloped, positive-sense single-stranded RNA virus belonging to the genus *Flavivirus* in the family *Flaviviridae*. It is an arthropod-borne virus characterized by neurotoxicity, with two major subtypes: the African and Asian lineages [29]. ZIKV is primarily transmitted through the bite of infected *Aedes* mosquitoes, but it can also spread via non-mosquito-mediated routes. Its genome, approximately 11 kb in length, encodes a polyprotein, which is processed by host and viral proteases into three structural proteins (capsid protein C, pre-membrane protein prM, and envelope protein E) and seven non-structural proteins (NS1, NS2A, NS2B, NS3, NS4A, NS4B, and NS5) [30,31]. After ZIKV enters the host cell, it replicates on the membranes of the endoplasmic reticulum, producing large amounts of double-stranded RNA, viral genomic RNA, and viral proteins. These viral components and hijacked host factors gather perinuclearly to form a viral replication complex, which supports efficient viral replication [32].

The G-quadruplex (G4) is a unique RNA secondary structure that plays a critical role at multiple stages of the viral lifecycle, such as transcription and translation [33,34]. ZIKV contains a highly conserved G4 structure within the stem-loop structure (3” SL) of its 3‘ untranslated region (3’ UTR), which is preserved across all ZIKV isolates and remains intact within the full-length 3‘ UTR transcript. RNA G-quadruplexes (rG4) are found not only in mRNA but also in non-coding RNA, participating in various essential cellular processes [35]. Studies have shown that DDX17, a newly identified rG4-binding protein, plays a role in G4-associated splicing and also mediates interactions between G4 and other proteins. Both DDX17 and DDX3X interact with the 3’UTR and can unwind the G4 structure in the 3’ SL in an ATP-dependent manner, thereby influencing ZIKV replication. This interaction presents a potential therapeutic approach for ZIKV infection by targeting G4-binding molecules or other compounds that disrupt the DDX17–3” SL interaction [36,37].

### DDX17 and rift valley fever virus (RVFV)

RVFV is a single-stranded negative-sense RNA virus primarily transmitted by mosquitoes, capable of causing severe illness and high mortality rates in both humans and livestock [38]. The genome of RVFV consists of three segmented, single-stranded negative-sense RNA segments: S, M, and L. The L and M segments are negative in polarity, with the L segment encoding an RNA-dependent RNA polymerase and the M segment encoding two envelope proteins and two accessory proteins [39]. The S segment exhibits ambisense coding, with the positive-sense RNA template encoding non-structural proteins, and the negative-sense RNA encoding the viral nucleoprotein [40]. The S segment includes a 5’ non-coding region (NCR) and an intergenic region (IGR), where DDX17 can inhibit RVFV replication by recognizing these non-coding regions. As an RNA helicase, DDX17 May unwind RVFV RNA via an ATP-dependent mechanism and interact with the structured viral RNA elements, thereby interfering with viral replication [41].

DDX17 demonstrates a dual role in recognizing RNA stem-loop structures. On the one hand, it binds to the stem-loop structure of host pri-miRNA, promoting its maturation; on the other hand, it binds to a crucial stem-loop structure within bunyavirus RNA, limiting viral infection. In Drosophila, the DDX17 homolog Rm62 has been shown to be an anti-RVFV helicase, with its absence exacerbating RVFV infection. Similarly, DDX17 and DDX5, two mammalian homologs of Rm62 [42], were tested in knockdown experiments, revealing that depletion of DDX17 significantly increased RVFV replication in U2OS cells, whereas DDX5 depletion did not show a comparable effect. This finding indicates a specific role of DDX17 in restricting RVFV replication [43]. A hallmark of the Bunyaviridae family is the presence of two hairpin structures within the S segment of the viral RNA, which serve as critical target motifs. In-depth CLIP-seq analysis has revealed interactions between DDX17 and the IGR and 5’ NCR regions of the RVFV S segment, indicating that DDX17 recognizes highly structured stem-loops within viral RNA [41]. Furthermore, DDX17 demonstrates evolutionary conservation in its role in inhibiting RVFV replication, with this inhibitory effect being independent of the interferon signaling pathway [43].

### DDX17 and human immunodeficiency virus type 1 (HIV-1)

HIV-1, the causative agent of AIDS, has a single-stranded positive-sense RNA genome comprising three structural protein genes (Env, Gag, and Pol), four accessory protein genes (Nef, Vif, Vpu, and Vpr), and two regulatory protein genes (Tat and Rev), encoding a total of 15 proteins [44]. HIV-1 relies on host proteins to complete its life cycle, with over 300 host proteins identified to facilitate its replication. As a cofactor of the zinc-finger antiviral protein (ZAP), DDX17 aids in the degradation of HIV-1 multiply spliced mRNA [45].

The HIV Rev protein is a critical regulatory factor in viral replication, especially in the late stages of the viral life cycle. Rev binds specifically to the Rev Response Element (RRE) in unspliced or partially spliced RNA transcripts, recruiting host proteins to form a complex that promotes export of the viral genome from the nucleus [46,47]. Studies have shown that DDX17 interacts with HIV-1 Rev protein and enhances the release of viral particles from cells [11].

Among the two isoforms of DDX17, the long form (p82) and the short form (p72), studies using a DQAD mutant of its DEAD domain have revealed that this mutant can reduce viral infectivity by blocking HIV-1 RNA packaging, significantly decreasing the amount of packaged viral RNA. Additionally, DDX17 can interact with the primary structural HIV-1 proteins Gag and Gag-Pol [48]. The DQAD mutant of DDX17 impairs Gag processing, and DDX17 regulates Gag-Pol frameshifting to support HIV-1 infection. Knockdown of DDX17 in HeLa cells using siRNA led to a twofold reduction in viral particle production, further confirming that DDX17 is essential for HIV-1 production [49].

HIV-1 lacks its own RNA helicase, depending instead on host helicases to facilitate various RNA-related processes. DDX17 and DDX5, which share 90% homology in their core domains and only 30% homology in their C-terminal domains, exhibit functional distinctions in biological processes [6]. Lever’s team screened a library of 59 human DDX helicases using siRNA and demonstrated that DDX17 and DDX5 act either as homodimers or heterodimers at multiple stages of the HIV-1 life cycle [50,51]. Further research indicated that DDX17, independently of DDX5, plays a central role in HIV-1 RNA splicing, utilizing its RNA-binding domain and C-terminal domain to facilitate RNA splicing, underscoring its critical role in viral replication. Although DDX17 and DDX5 are highly homologous, they exhibit functional divergence in the HIV-1 replication cycle, with DDX5 unable to perform DDX17’s crucial role in regulating the production of spliced HIV mRNA. This unique function of DDX17 highlights its potential as a therapeutic target for HIV treatment [52].

### DDX17 and hepatitis B virus (HBV)

HBV is a non-cytopathic hepatotropic virus that, upon infection, can lead to hepatitis, liver fibrosis, cirrhosis, and even hepatocellular carcinoma [53]. A unique reverse transcription process characterizes HBV replication: after its relaxed circular DNA (rcDNA) is released into the host cell nucleus, it forms covalently closed circular DNA (cccDNA), establishing a basis for the virus’s long-term persistence and replication [54,55]. cccDNA serves as the sole template for all viral mRNA synthesis, thus acting as a molecular reservoir for HBV’s persistent infection [56,57].

RNA helicases DDX17 and DDX5 play crucial roles in regulating gene expression, particularly by fine-tuning the accuracy of gene expression through coupling transcription with co-transcriptional mRNA processing. These proteins are also critical regulators in recognizing the HBV 3” polyadenylation signal (cPAS) [58,59]. In various cell models, DDX17 and DDX5 are essential for transcription termination and mRNA 3” end processing [60,61]. Using immunoprecipitation assays with antibodies against DDX5 or DDX17, Chapus and colleagues found that both proteins associate with cccDNA and RNA, suggesting their direct role in cPAS recognition and thus in regulating HBV 3’ mRNA processing [62].

Knockout of DDX17, as well as preferential cPAS recognition, correlates closely with enhanced HBV replication, revealing a functional link between transcription termination mechanisms and normal HBV replication. Studies have shown that most HBV RNAs terminate transcription 14 nucleotides downstream of cPAS, though some transcripts exhibit read-through across approximately 700 nucleotides. DDX5 and DDX17 facilitate this read-through by blocking cPAS recognition, playing a critical role in HBV mRNA 3’ end processing, a process that supports viral replication [62].

HB viral protein X (HBx), a multifunctional regulatory protein essential for HBV infection, is encoded by the HBx transcript, and HBV read-through destabilizes the HBx transcript [63,64]. DDX17 and DDX5 promote transcriptional read-through by inhibiting specific cPAS recognition, thus suppressing HBx expression. DDX17 plays a vital role in post-transcriptional regulation by modulating HBx mRNA stability, thereby indirectly promoting effective HBx expression. When DDX17 is overexpressed, HBx levels rise significantly; conversely, HBx expression is markedly reduced when DDX17 is depleted. HBx can upregulate DDX17, further enhancing HBV transcription and replication.

Studies also indicate that depletion of ZWINT significantly reduces HBV RNA and DNA levels, inhibiting HBV transcription and replication. DDX17 indirectly promotes HBV replication by upregulating ZWINT expression [65]. Additionally, DDX17 acts as an intrinsic restriction factor for HBV, primarily by blocking the glycosylation of HBV pgRNA, thereby suppressing replication. The pgRNA contains redundant stem-loop structures, termed ε, at its 5’and 3’ ends. DDX17 binds to the ε structure on pgRNA, blocking its glycosylation in a helicase-dependent manner. This mechanism could provide a novel therapeutic target for developing HBV treatments [15].

### DDX17 and Hantaan virus (HTNV)

HTNV is an enveloped zoonotic virus with a single-stranded, negative-sense RNA genome, primarily transmitted through rodents. Its tripartite genome is segmented into L, M, and S fragments, where the L segment encodes the RNA-dependent RNA polymerase (RdRp), the M segment encodes the glycoprotein precursor (GPC), and the S segment encodes the nucleocapsid protein (NP) [66]. HTNV encodes one NP, which forms the ribonucleoprotein complex (RNP) with viral polymerase, serving as both an active template for RNA synthesis and a structure packaged into viral particles [67–69].

The 3’ and 5’ ends of the HTNV RNA genome contain highly conserved and complementary sequences that form a panhandle-like secondary structure. During viral replication, GPC is cleaved into two glycoproteins, Gn and Gc. Co-expression of viral genome RNA and NP stabilizes Gn by protecting it from autophagic degradation, which is essential for HTNV replication. The accumulation of NP, Gc, and the viral S segment RNA collectively stabilizes Gn, thereby supporting viral replication and proliferation [70–72].

HTNV mRNA synthesis relies on a unique cap-snatching mechanism to initiate viral mRNA synthesis and genome replication, using host-derived 52019;-capped sequences [73]. The interaction between NP and RdRp enables RdRp to capture 52019;-capped RNA fragments from host cell transcripts, which are then used as primers to initiate transcription and replication of the viral genome in the cytoplasm of infected cells. NP also binds to the 52019; cap of host mRNA, shielding it from cellular decapping processes [74].

Research has shown that HTNV infection modulates DDX17 expression in a biphasic manner. Western blot analysis indicates that DDX17 levels increase significantly in the early stages of HTNV infection but decrease at later stages. Overexpression of DDX17 promotes HTNV replication overall. Upon HTNV infection, NP competes with DDX17 for nuclear-cytoplasmic transport molecules, leading to DDX17 translocation from the nucleus to the cytoplasm. Further investigation reveals an interaction between DDX17 and HTNV NP, specifically between the helicase domain of DDX17 and the RNA-binding domain of NP. This interaction enhances the unwinding of the RNP complex and facilitates cap acquisition, thereby promoting HTNV replication [13].

### DDX17 and influenza a virus (IAV)

Influenza virus, particularly IAV, is a major pathogenic threat to human health, frequently causing seasonal outbreaks with high morbidity and mortality rates [75,76]. IAV is an enveloped, single-stranded negative-sense RNA virus with a genome consisting of eight distinct negative-sense RNA (vRNA) segments, which form viral ribonucleoprotein complexes (vRNPs) [77]. The IAV RNA-dependent RNA polymerase (RdRp) is a trimeric complex composed of polymerase basic protein 1 (PB1), polymerase basic protein 2 (PB2), and polymerase acidic protein (PA) [78]. PB1 primarily functions as the RNA polymerase, PB2 recognizes and binds to the cap structure of host mRNA, and PA contains an endonuclease domain [79].

The NP protein, an essential component of vRNP, is crucial for maintaining IAV polymerase activity. NP-induced mitophagy serves as a novel regulatory mechanism during IAV infection, allowing the virus to evade host antiviral responses and enhance replication. Despite encoding multiple proteins, IAV requires host proteins for efficient replication [80–82]. Studies have demonstrated that in H5N1 subtype AIV-infected cells, DDX17, as a host protein, interacts with NP and acts as a significant regulator of H5N1 influenza virus replication within host cells. DDX17 functions in both human and avian cells infected with H5N1, facilitating the efficient synthesis of viral mRNA and vRNA. In the early stages of H5N1 infection, DDX17 colocalizes with NP in the nucleus, suggesting its involvement in viral RNA synthesis. In later stages, DDX17 and NPM1 associate with cytoplasm-exported vRNPs, redistributing alongside NP in the cytoplasm, which indicates that vRNA may be transported from the nucleus to the cytoplasm for further replication or packaging [83,84].

Experiments in A549 cells where DDX17, DDX5, or DDX3 were knocked down showed that these helicases modulate viral polymerase activity, with a significant decrease in viral titers observed upon knockdown [12]. These findings suggest that DDX17 serves as a crucial host factor in influenza virus replication and may represent a potential therapeutic target for anti-influenza drugs.

### Role of DDX17 in other viruses

In late December 2019, an outbreak of COVID-19 occurred in China, caused by the novel coronavirus SARS-CoV-2. Characterized by high infectivity, rapid transmission, and acute onset, COVID-19’s primary symptoms include fever, fatigue, and dry cough. Severe cases may progress to acute respiratory distress syndrome (ARDS), septic shock, uncorrectable metabolic acidosis, coagulation dysfunction, and even multi-organ failure [85–87]. SARS-CoV-2 is a novel β-coronavirus with a positive-sense single-stranded RNA genome encoding two polyprotein precursors (pp1a and pp1ab), four structural proteins (S, E, M, and N), and several accessory proteins [88].

In anti-COVID-19 research, DDX17 has been identified as a potential host gene affected by infection. Studies have shown that natural compounds such as apigenin, quercetin, and resveratrol can influence key genes like DDX17, suggesting that these three agents may be potential therapeutic candidates against COVID-19 infection [89].

Additionally, DDX17 plays important roles in other viral infections. For example, Epstein – Barr virus (EBV) is a DNA virus associated with various human cancers, including malignant lymphomas, nasopharyngeal carcinoma, and gastric carcinoma, primarily infecting human lymphocytes and oropharyngeal epithelial cells [90–92]. N6-methyladenosine (m6A) modification, by influencing RNA metabolism, can inhibit EBV’s lytic replication. YTHDF1, YTHDF2, and YTHDF3 are prominent “reader” proteins that regulate RNA metabolism based on m6A signaling [93,94]. Studies have revealed that DDX17 and ZAP interact with YTHDF1, and their knockdown can enhance EBV infection and replication. DDX17, as an essential component of the RNA decay complex, interacts with YTHDF1, promoting viral RNA decapping efficiency and mediating RNA decay [16].

Human T-cell leukemia virus type 1 (HTLV-1) is a retrovirus closely linked to human T-cell leukemia [95]. Tax and HBZ are two critical genes in HTLV-1’s immune evasion; Tax is required for viral replication and activates various signaling pathways, while HBZ inhibits apoptosis and influences hematopoietic stem cell differentiation [96–98]. NF-κB is a widely expressed transcription factor responsive to external stimuli, including radiation and viral infection, and is involved in cellular inflammation and immune responses [99,100]. Studies indicate that Tax dynamically coordinates the interaction between the transcription factor RELA and the splicing regulator DDX17 by activating the NF-κB pathway. Tax-induced NF-κB leads to significant RELA enrichment in the genome, recruiting splicing factor DDX17, which, through its RNA helicase activity, modulates the alternative splicing of CD44 to promote ATLL progression [101]. In ATL-2 and Jurkat-HBZ cells, HBZ has been found to partially colocalize and interact with DDX5 and DDX17, further underscoring their roles in the viral pathogenic mechanism [102].

## Summary

DDX17 is an RNA helicase widely present in eukaryotic cells, involved in multiple RNA metabolic processes such as RNA splicing, transcriptional regulation, nuclear export, and post-transcriptional regulation. Recent studies have highlighted DDX17’s multifaceted roles as a host factor in viral replication, revealing its dual function across various viral infections. DDX17 can either enhance viral replication or inhibit viral proliferation by interfering with viral RNA metabolism, underscoring its critical regulatory role in host-virus interactions (Table 2).

**Table 2.** The interaction between DDX17 and viruses.

Virus	Role/Interaction sites
SINV	Promotes viral infection; co-inhibition with DDX5 exhibits cumulative antiviral effects [28]
ZIKV	Interacts with the 32019; UTR [36,37]
RVFV	Inhibits RVFV replication; interacts with the stem-loop structure at the 5’ end of the S segment [41,43]
HIV-1	Promotes Rev-dependent export of HIV RNA and production of infectious HIV-1 particles [48,49]; degrades HIV-1 mRNA [45]
HBV	Positively correlates with HBV replication; interacts with HB viral protein X (HBx) [65]
HTNV	Interacts with HTNV NP to promote HTNV replication [13]
IAV(H5N1)	Interacts with NP [83]; required for H5N1 RNA synthesis and enhances influenza virus replication [83,84]
COVID-19	Gene potentially affected by COVID-19 infection [89]
EBV	Inhibits EBV infection and replication; promotes viral RNA decapping and mediates RNA decay [16]
HTLV-1	Partially co-localizes and interacts with HBZ [102]

### Mechanisms promoting viral replication

In some viral infections, DDX17 facilitates viral replication by interacting with viral and host proteins, enhancing the stability and packaging efficiency of viral RNA. For instance, DDX17 interacts with HIV-1’s Rev protein, promoting the release of viral particles from host cells and playing essential roles at different stages of viral replication. Studies have also demonstrated that DDX17’s helicase function enhances the packaging efficiency of HIV-1 viral RNA by regulating Gag polyprotein processing, thereby increasing viral infectivity [49].

During influenza virus infection, DDX17 interacts with the viral NP protein and promotes viral RNA synthesis by regulating polymerase activity. In H5N1-infected cells, DDX17 serves as a critical host factor that regulates the synthesis of viral mRNA and vRNA, thereby enhancing viral replication [12]. Additionally, in Hantaan virus (HTNV) infection, DDX17 interacts with the viral NP protein, facilitating viral cap-snatching, which enhances the unwinding efficiency of the viral RNP complex, ultimately promoting viral replication and dissemination [13].

### Mechanisms inhibiting viral replication

Conversely, DDX17 exhibits an inhibitory effect in certain other viral infections. For example, in HBV infection, DDX17 interferes with viral RNA processing at the 3’ end by blocking glycosylation, thereby inhibiting HBV replication. Through interaction with HBV’s cPAS, DDX17 regulates viral RNA processing and termination, preventing transcriptional readthrough and thus impacting HBV gene expression [62].

In Epstein-Barr virus (EBV) infection, DDX17 interacts with the m6A “reader” protein YTHDF1 to promote viral RNA decapping and mediate RNA degradation, thus inhibiting EBV replication. Knockdown of DDX17 leads to viral RNA accumulation, increasing EBV infectivity and replication. These findings indicate that DDX17 acts as a host restriction factor, playing an antiviral role by regulating decapping and degradation of viral RNA [16].

### Dual roles of DDX17 and its regulatory mechanisms

The dual roles of DDX17 across different viral infections are closely related to its unique structural functions. As a DEAD-box RNA helicase, DDX17 possesses a highly conserved core domain, while its C-terminal domain is more variable, granting DDX17 versatility and specificity in interacting with distinct viral proteins. For instance, in HIV-1 infection, DDX17 promotes viral RNA packaging and stability through its core helicase domain interactions with viral proteins; in HBV infection, its C-terminal domain is involved in RNA termination regulation, restricting viral proliferation [52,62].

The regulatory mechanisms behind this dual functionality suggest that DDX17 does not simply act as a pro- or anti-viral factor; rather, its role depends on the specific viral type, infection stage, and host cellular environment. This flexibility provides new insights into the complex interplay between host and virus, and also identifies potential targets for antiviral drug development.

### Potential of DDX17 as an antiviral target

Given DDX17’s pivotal role in various viral infections, it holds significant potential as a target for antiviral therapy. DDX17 is involved in viral RNA packaging and replication, as well as gene expression regulation through RNA splicing and decapping. Developing specific inhibitors or small molecules targeting DDX17’s regulatory mechanisms could effectively disrupt the viral replication cycle, thereby limiting viral spread and infection.

For example, in HIV-1 infection, DDX17 promotes viral particle release by interacting with the Rev protein. Designing specific small-molecule inhibitors to block the DDX17-Rev interaction could effectively suppress HIV-1 replication. Similarly, in influenza virus and HTNV infections, DDX17’s interaction with viral RNP complexes is critical for viral RNA synthesis and packaging. Thus, disruptors targeting these interactions could provide novel antiviral therapeutic strategies.

On the other hand, DDX17’s inhibitory role in viral replication is equally noteworthy. In HBV and EBV infections, DDX17 limits viral replication by blocking viral RNA glycosylation and promoting decapping. Enhancing DDX17 expression or activity to strengthen its antiviral functions may represent a promising new antiviral strategy.

## Conclusion and recommendations

With advancements in molecular biology, researchers have gained a deeper understanding of the multifaceted roles of DDX17 in viral infections. DDX17 plays pivotal roles not only in RNA metabolism and antiviral immunity but also in regulating the viral replication cycle through interactions with viral and host proteins, underscoring its significance as a critical host factor. However, current knowledge about the mechanisms and virus-specific regulatory roles of DDX17 remains limited. In particular, further exploration is needed to clarify the dynamic functions of DDX17 under diverse viral infection conditions and integrate these findings into a comprehensive mechanistic framework.

Future studies should focus on several key areas: first, unraveling the molecular basis of DDX17 interactions with viral proteins to determine its precise regulatory mechanisms during the viral replication cycle. Second, the dual roles of DDX17 in viral infections – facilitating or suppressing replication – suggest a need to investigate strategies for selectively modulating its activity or expression to target specific viral replication processes. Lastly, as a potential antiviral therapeutic target, DDX17 holds significant clinical promise. Developing specific inhibitors or small-molecule drugs to disrupt critical interactions between DDX17 and viral components could pave the way for innovative antiviral strategies.

Unlike previous reviews focusing solely on DDX17’s pro- or anti-viral roles, this study systematically compares its context-dependent functions. For example, DDX17’s interaction with viral RNA structures (e.g. G-quadruplexes in ZIKV) versus host RNA processing machinery (e.g. cPAS in HBV) suggests a bifurcated regulatory mechanism. Future studies should explore whether targeting these distinct interactions could yield virus-specific therapeutics.

Furthermore, emerging evidence suggests that DDX17 also contributes to epithelial-mesenchymal transition (EMT) and tumor progression across multiple cancer types. For instance, DDX17 promotes EMT in breast cancer by modulating alternative splicing of CD44, thereby enhancing metastatic potential [103]. These findings expand the functional scope of DDX17 beyond viral infections and highlight its broader implications in cellular transformation.

In summary, research on DDX17 not only enhances our understanding of virus-host interactions but also points toward promising directions for future antiviral therapeutic development. As investigations progress, DDX17 May emerge as a key target for combating viral infections, offering new hope for mitigating the burden of virus-associated diseases and contributing substantially to human health and public welfare.

## Data sharing statement

Data sharing does not apply to this article, as no new data were created or analyzed in this study.
